# MicroRNA-486-5p functions as a diagnostic marker for carotid artery stenosis and prevents endothelial dysfunction through inhibiting inflammation and oxidative stress

**DOI:** 10.1080/21655979.2022.2054500

**Published:** 2022-03-24

**Authors:** Bin Zhu, Wei Liu, Qiang Xu, Hong-Liang Liu

**Affiliations:** Department of Neurosurgery, Renhe Hospital, Baoshan District, Shanghai, China

**Keywords:** Carotid artery stenosis, miR-486-5p, endothelial dysfunction, inflammation, oxidative stress

## Abstract

Carotid artery stenosis (CAS) can cause ischemic stroke, and clinical intervention for CAS is critical clinically. The purpose of this study was to explore the expression changes of microRNA-486-5p in the serum of patients with CAS and its possible mechanism. Ninety-one cases with asymptomatic CAS were recruited, and serum levels of miR-486-5p were measured using RT-qPCR. The diagnostic ability was evaluated by drawing the receiver operating characteristic (ROC) curve. Human aortic endothelial cells (HAECs) were treated with oxidized low-density lipoprotein (oxLDL) to establish cell model, and cell proliferation and apoptosis were tested. The markers of cell inflammation and oxidative stress were detected via ELISA. The target gene was analyzed using bioinformatics analysis combined with luciferase reporting assay. CAS cases exhibited significantly low serum miR-486-5p levels in comparison with the control group and can identify asymptomatic CAS. Serum miR-486-5p manifested a negative correlation with the degree of carotid stenosis. Underexpression of miR-486-5p was also detected in ox-LDL treated HAECs. OxLDL treatment contributes to inflammatory response and oxidative stress of HAECs; however, these adverse impacts caused by ox-LDL were reversed by miR-486-5p upregulation. NFAT5 was confirmed to be the target gene of miR-486-5p in HAECs. MiR-486-5p serves as a promising biomarker for the early identification of CAS. Overexpression of miR-486-5p can prevent endothelial dysfunction, and the mechanism might be related to anti-inflammation and anti-oxidation via targeting NFAT5.

## Graphical abstract

**Figure:**
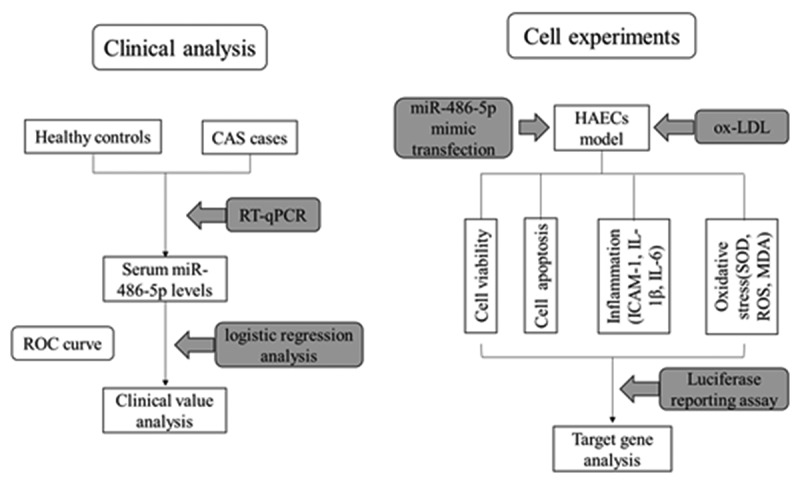

## Introduction

Ischemic cerebrovascular disease is a kind of disease that severely affects life safety. It is characterized by high incidence, disability rate, fatality rate, and recurrence rate. Carotid artery stenosis (CAS) can reflect the degree of systemic arteriosclerosis and is one of the factors of ischemic stroke [1]. The main cause of CAS is the dysfunction of endothelial cells caused by atherosclerotic plaque, and the apoptosis of vascular endothelial cells induced by oxidized low-density lipoprotein (ox-LDL) is a vital link leading to the lesion [2]. It is demonstrated that age, hypertension, diabetes, and hyperlipidemia are all risk inducements for CAS [3]. Clinical intervention for asymptomatic CAS patients can effectively reduce the risk of stroke, which has an important clinical significance.

In recent years, with the development of gene diagnostic technology, the role of microRNAs (miRNAs) in various diseases has attracted attention [^4–6^]. Studies have shown that there are differential expression profiles of miRNAs in patients with CAS, suggesting the potentially important role of miRNAs in CAS [^7–9^]. MiRNA is a kind of non-coding small molecule with 17 ~ 22 nucleotides in length, and its dysregulation is related to the occurrence and development of a variety of diseases [10,11]. It can bind to the 3’-untranslated regions of the specific target genes and then degrade or inhibit the mRNA post-translational levels of the target genes [12]. Due to the stable expression of miRNA in peripheral blood, miRNA has become a biomarker of many diseases in recent years [^13–15^]. MiR-486-5p is one of the important miRNAs with high abundance expression in the heart, downregulation of miR-486-5p has been detected in coronary atherosclerotic plaques via microarray experiments [16]. In addition, significantly reduced miR-486-5p is detected in individuals with high blood pressure, which shows a significant association with the cardiovascular risk score [17]. These results demonstrate the important influence of miR-486-5p on arteriosclerosis, which encourages us to explore its role in CAS.

In this study, a real-time quantitative reverse transcription-polymerase chain reaction (RT-qPCR) combined with bioinformatics analysis was used to investigate the expression changes of miR-486-5p in the serum of patients with CAS, and its clinical value was evaluated. In addition, human aortic endothelial cells (HAECs) were treated with oxidized low-density lipoprotein (oxLDL) to establish cell models, and the regulatory role of miR-486-5p in cell proliferation, apoptosis, cell inflammation, and oxidative stress was detected. It is attempted to lay a foundation for the further construction of the molecular regulatory network of miRNA in CAS.

## Materials and methods

Ninety-one cases with asymptomatic CAS and 87 healthy people were included, and serum miR-486-5p levels were detected using real-time quantitative polymerase chain reaction (RT-qPCR). HAECs were treated with oxLDL, and miR-486-5p levels were regulated in vitro. Cell proliferation and apoptosis were detected using a cell counting kit-8 (CCK-8) assay and a flow cytometry assay. Cell inflammation and oxidative stress-related factors were detected through an enzyme-linked immunosorbent assay (ELISA) assay. A double luciferase reporting assay was applied for the target gene analysis.

### Study subjects and blood specimen collection

The CAS group consisted of 91 cases who underwent physical examination and were diagnosed with asymptomatic CAS via carotid artery ultrasound in the Department of Neurology of Renhe Hospital. The inclusion criteria are as follows: (1) aged more than 50 years old; (2) the degree of carotid artery stenosis was confirmed by carotid ultrasound examination to be >50%; (3) no history of ischemic stroke, focal neurological symptoms, transient ischemic attack or amaurosis was found by inquiring medical history; (4) was consciousness and cooperative with the physical examination; (5) the study was reviewed and approved by the Ethics Committee of Renhe Hospital [No. HIRB190017], and all subjects signed informed consent. The exclusion criteria are as follows: (1) with ischemic or hemorrhagic stroke; (2) accompanied with a malignant tumor; (3) accompanied with immune system diseases; (4) have a mental illness in the past and cannot cooperate with the examination; (5) accompanied with heart, liver, and renal dysfunction; and (6) family history of hereditary diseases. In addition, 87 healthy people who underwent physical examination in Renhe Hospital during the same period were selected as the control group. 10 ml elbow venous blood was taken from each participant and centrifuged at 3000 rpm for 20 min. The serum samples were collected and kept in the refrigerator at −80°C for use. 5 ml of blood was used for the detection of blood biochemical indexes, and the rest of the blood was used for the qRT-PCR.

### Cell culture and transfection

Human aortic endothelial cells (HAECs) were provided by the American Type Culture Collection (ATCC), and endothelial cell medium (ECM) was used for the cell culture combined application of 5% fetal bovine serum (FBS). The cells were incubated under the circumstance of 5% CO_2_ at 37°C. When the cells grow to 90% confluency, the cell transfection was done to regulate the mRNA expression in cells. Sequences of miR-486-5p mimic or inhibitor or their negative controls (mimic-NC and inhibitor NC) were synthesized and provided by the Tiangen Biological Company and transiently transfected into HAECs using lipofectamine 2000. Then, the HAECs were treated with 20 µg/ml oxidized low-density lipoprotein (oxLDL, Guangzhou Yiyuan Biological Technology Co., Ltd., Guangzhou, China) for 48 h [18].

### RT-qPCR

TRIzol was applied to separate and extract total RNA, and Nanodrop 2000 was used to measure RNA concentration. QuantiMiR RT Kit (Systems Biosciences) and Script II Reverse transcriptase (Invitrogen) reverse transcription kit (Invitrogen) were used to transcribe the miRNA or mRNA into cDNA. The relative mRNA expression level was detected by 2× SYBR Green Master Mix (Applied Biosystems) in Applied Biosystems Model 7500 qRT-PCR amplifier. The relative expression of miR-486-5p and the nuclear factor of activated T cell 5 (NFAT5) was calculated using the 2 ^−ΔΔCt^ method via using U6 and GAPDH as the reference genes, respectively. All primers were designed and synthesized by Sangon (Shanghai, China) and the sequences were as follows: miR-486-5p, forward 5’-GAATTTGGAGTTTAGTTATAGTTTTTATT-3’ and reverse 5’-CCCAACACCACACACACCATACTA-3’; U6, forward 5’-CTCGCTTCGGCAGCACA-3’ and reverse 5’-AACGCTTCACGAATTTGCGT-3’; NFAT5, forward 5’-ACCCAGAGACCCTGACAACT-3’ and reverse 5’-TGAAACTGGGTAGCCTGCTG-3’; GAPDH, forward 5’-GGAGGGCCTCATGACCACCGT-3’ and reverse: 5’-CACATCTTCCCAGAGGGGCCGT-3’.

### CCK-8 assay

CCK-8 assay was done to evaluate the cell viability of the cells. The cell viability was monitored for three consecutive days, and 10 µL of CCK-8 solution was added to each well at 0, 24, 48, and 72 h, respectively. After 2 hours of incubation at 37°C, the optical density (OD) value was measured at 450 nm with a microplate reader.

### Flow cytometry assay

To detect cell apoptosis, the cells were stained with Annexin V-FITC/Propidium Iodide (PI) detecting kits (Nanjing KeyGen Biotech, Nanjing, China) by flow cytometry based on the manufacturer’s protocol. 1 mL of cell suspension was taken and centrifuged, precipitation was collected, and 5 μL Annexin V-FITC and PI were added, respectively, and incubated for 30 min in the dark. The cell apoptosis rate of different groups was measured by flow cytometry, and the experiments were repeated 3 times.

### ELISA assay

After centrifugation, the supernatant of each cell group was collected. The concentration of interleukin-6 (IL-6), IL-1β, soluble intercellular adhesion molecule-1 (sICAM-1), superoxide dismutase (SOD), reactive oxygen species (ROS), and malondialdehyde (MDA) in the cell culture medium was determined according to the instructions of the enzyme-linked immunosorbent assay (ELISA) kit.

### Double luciferase reporting assay

TargetScan was used for the target gene analysis, it was found that there were binding sites in the 3’-untranslated region (3’-UTR) of miR-486-5p and NFAT5. The wild type (Wt) and mutant type (Wt) NFAT5 luciferase reporter gene vectors were constructed, respectively. After the above two vectors were mixed with miR-486-5p mimic and miR-486-5p inhibitor, respectively, they were co-transfected into HAECs. After 48 hours of culture, the cells were collected and washed with phosphoric acid buffer (PBS) to detect luciferase activity.

### Statistical analysis

Experimental data are expressed by mean ± standard deviation (SD). Spss 22.0 and GraphPad 7.0 software were used for statistical analysis. Student's t-test and one-way ANOVA analysis were used to assess the difference among different groups. The diagnostic ability was evaluated by drawing the receiver operating characteristic (ROC) curve, and the diagnostic sensitivity and specificity were calculated. The influence of clinical indicators on the occurrence of disease was detected by logistic regression analysis. *P* < 0.05 means the difference is statistically significant.

## Results

### Clinical indicators of the study population

Clinical indicators of the study groups are shown in Table 1. We discovered that the CAS patients possessed significantly high levels of low-density lipoprotein (LDL), systolic blood pressure (SBP), diastolic blood pressure (DBP), C-reactive protein (CRP), and soluble intercellular adhesion molecule-1 (sICAM-1) in contrast to the healthy people (*P* < 0.001). However, no significant difference emerged with respect to other indicators between the two groups (*P* > 0.05), including age, gender, body mass index (BMI), fasting blood glucose (FBG), total cholesterol (TC), triglycerides (TG), and high-density lipoprotein (HDL).

**Table 1.** Comparison of indicators among the study population

Parameters	Controls (n = 87)	Patients (n = 91)	*P* value
Gender			0.740
Male	49	49	
female	38	42	
Age (years)	63.83 ± 6.80	63.18 ± 7.12	0.533
BMI (kg/m^2^)	23.07 ± 2.62	22.67 ± 3.02	0.353
FBG (mg/dL)	89.67 ± 16.32	92.80 ± 16.70	0.209
TC (mg/dL)	191.74 ± 4.86	192.59 ± 4.29	0.221
TG (mg/dL)	121.61 ± 13.22	125.24 ± 12.99	0.066
HDL (mg/dL)	49.71 ± 3.75	49.05 ± 4.16	0.268
LDL (mg/dL)	108.52 ± 5.73	110.39 ± 6.74	0.048
SBP (mm Hg)	122.46 ± 6.26	124.95 ± 9.13	0.036
DBP (mm Hg)	73.93 ± 5.83	89.41 ± 5.00	<0.001
CRP (mg/L)	5.03 ± 2.10	24.91 ± 3.48	<0.001
sICAM-1 (ng/mL)	442.52 ± 78.95	576.60 ± 141.78	<0.001

Acronym interpretation: BMI, body mass index; FBG, fasting blood glucose; TC, total cholesterol; TG, triglycerides; HDL, high-density lipoprotein; LDL, low density lipoprotein; SBP, systolic blood pressure; DBP, diastolic blood pressure; CRP, C-reactive protein; sICAM-1, soluble intercellular adhesion molecule-1. Data are expressed as n or mean ± standard deviation.

### Aberrant expression of miR-486-5p in CAS and its diagnostic ability analysis

**Figure 1.**
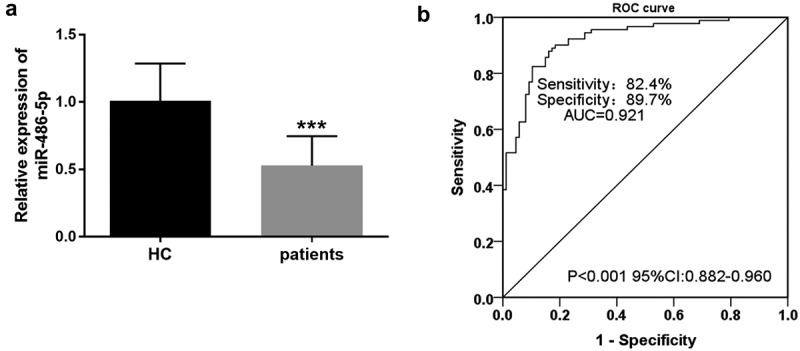
The diagnostic value of dysregulation of miR-486-5p in CAS patients. A. CAS cases exhibited significantly low serum miR-486-5p levels when compared with the control group (*P* < 0.001). B. ROC curve of serum miR-486-5p for identifying CAS from healthy individuals.

As shown in (Figure 1a), CAS cases exhibited significantly low serum miR-486-5p levels when compared with the control group (*P* < 0.001). Furthermore, we performed an ROC analysis to assess the diagnostic ability of miR-486-5p for CAS. As seen from Figure 1b, the area under the curve (AUC) of miR-486-5p was 0.921 (95% confidence interval (CI) = 0.882–0.960, *P* < 0.001). Then, the Youden index was performed to calculate the best cutoff value. The cutoff value of 0.692 carries the maximum Youden index, and the corresponding sensitivity and specificity were 82.4% and 89.7%, respectively.

### Association of serum miR-486-5p with clinical indicators

Pearson’s correlation analysis was done for the correlation evaluation. Table 2 shows a negative correlation of serum miR-486-5p with LDL (r = −0.521), SBP (r = −0.261), DBP (r = −0.272), CRP (r = −0.325), sICAM-1 (r = −0.424), as well as degree of carotid stenosis (r = −0.680). In view of the aberrant expression of miR-486-5p in CAS patients and the significant correlation with the degree of carotid stenosis, the logistic regression analysis was further performed to estimate its influence on CAS degree after adjusting other clinical factors. We can observe from Table 3 that miR-486-5p had independent influence on the degree of carotid stenosis (odds ratio (OR) = 0.236, 95% CI = 0.079–0.704, *P* = 0.010).

**Table 2.** Correlation between miR-486-5p and clinical indicators

Characteristics	r
LDL	−0.521**
SBP	−0.261*
DBP	−0.272*
CRP	−0.325**
sICAM-1	−0.424**
Degree	−0.680**

Acronym interpretation: r, correlation between miR-486-5p and various indicators; LDL, low density lipoprotein; SBP, systolic blood pressure; DBP, diastolic blood pressure; CRP, C-reactive protein; sICAM-1, soluble intercellular adhesion molecule-1. ** means significant correlation at the 0.01 level (two-sided); * means significant correlation at the 0.05 level (two-sided).

**Table 3.** Association of different variables with degree of carotid artery stenosis

Variables	OR	95% CI	*P* value
MiR-486-5p	0.236	0.079–0.704	0.010
Gender	1.126	0.400–3.169	0.822
Age	1.446	0.425–4.915	0.555
BMI	1.427	0.510–3.991	0.498
FBG	1.610	0.531–4.876	0.400
TC	1.858	0.607–5.690	0.278
TG	0.872	0.311–2.443	0.794
HDL	0.596	0.204–1.746	0.346
LDL	2.036	0.677–6.126	0.206
SBP	1.837	0.667–5.057	0.239
DBP	1.903	0.664–5.449	0.231
CRP	2.333	0.803–6.779	0.120
sICAM-1	2.066	0.704–6.066	0.187

Acronym interpretation: BMI, body mass index; FBG, fasting blood glucose; TC, total cholesterol; TG, triglycerides; HDL, high-density lipoprotein; LDL, low density lipoprotein; SBP, systolic blood pressure; DBP, diastolic blood pressure; CRP, C-reactive protein; OR, odds ratio; 95% CI, 95% confidence interval.

### The negative regulatory effect of miR-486-5p on the cell apoptosis, inflammation, and oxidative stress of HAECs

**Figure 2.**
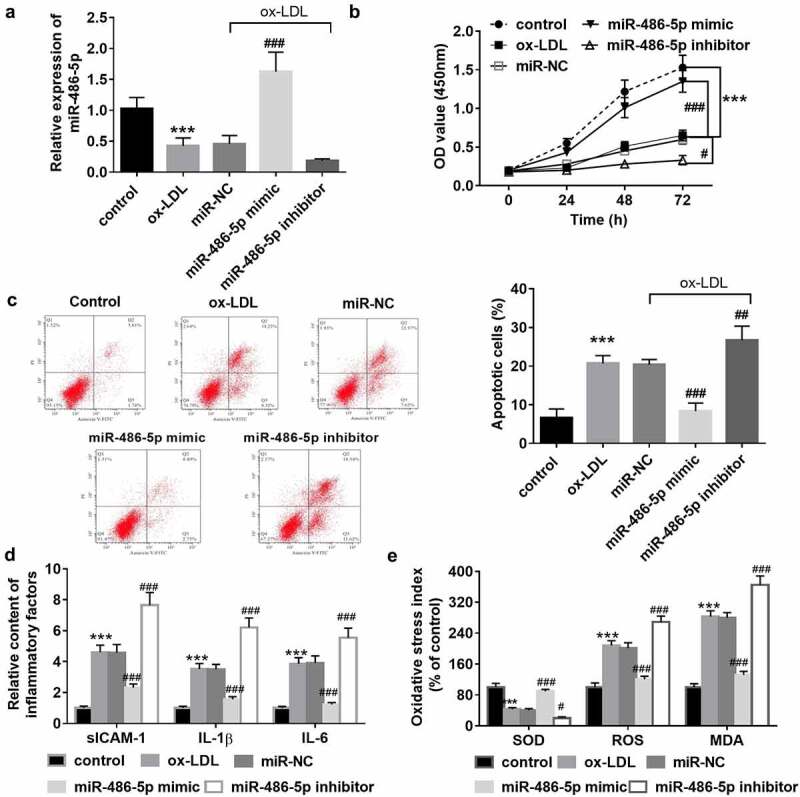
In ox-LDL treated cells, low levels of miR-486-5p were also detected, which was reversed by miR-486-5p mimic transfection (a). MiR-486-5p negatively regulated the HAECs apoptosis (b), inflammation (c) and oxidative stress (d) induced by ox-LDL. *** *P* < 0.001 (VS control group); ^###^ *P* < 0.001 (VS ox-LDL group).

Ox-LDL was applied for the HAECs to mimic the endothelial dysfunction caused by atherosclerosis. After ox-LDL induction, the cell proliferation was suppressed (Figure 2b), while the cell apoptosis was promoted (Figure 2c). Also, markers of the inflammatory response (Figure 2d) and oxidative stress (Figure 2e) were successfully induced. In ox-LDL treated cells, underexpression of miR-486-5p was also detected, and it was consistent with its levels in clinical serum samples (Figure 2a). Then, cell transfection was performed for the miR-486-5p level regulation to explore the role of miR-486-5p in cell function. As observed from Figure 2b-c, the overexpression of miR-486-5p can promote HAEC proliferation, while suppressing cell apoptosis. Besides, ox-LDL induced release of inflammatory factors and oxidative stress indicators was reversed by miR-486-5p (Figure 2d-e). But miR-486-5p inhibitor transfection further exacerbated the cell apoptosis, inflammation, and oxidative stress of HAECs induced by ox-LDL (Figure 2b-e).

### Target relationship confirmation between miR-486-5p and NFAT5

**Figure 3.**
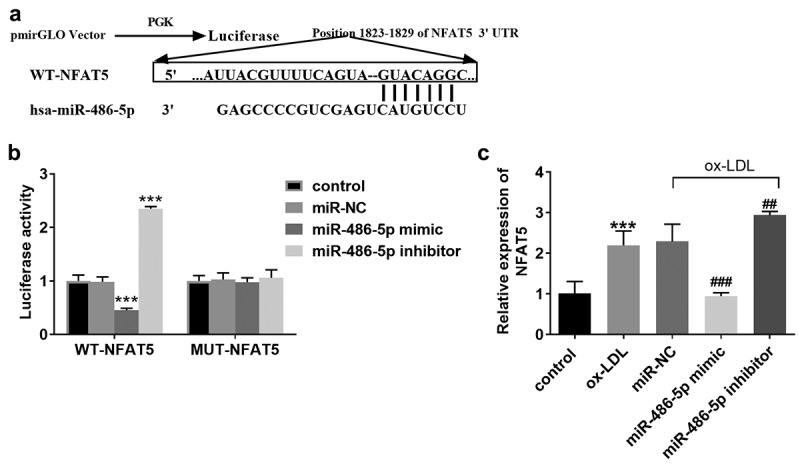
Target relationship confirmation between miR-486-5p and NFAT5. A. TargetScan showed a target binding sequence between miR-486-5p and NFAT5. B. The luciferase activity of HAECs was regulated by miR-486-5p mimic or inhibitor. C. The mRNA levels of NFAT5 in ox-LDL treated cells transfected with miR- miR-486-5p mimic or inhibitor. *** *P* < 0.001 (VS control group); ^###^ *P* < 0.001 (VS ox-LDL group).

Figure 3a shows the target-binding sequence between miR-486-5p and NFAT5 through TargetScan analysis. The luciferase reporter assay showed that miR-486-5p mimic transfection led to the reduction of cell luciferase activity, whereas miR-486-5p inhibitor transfection contributed to the increase of luciferase activity (Figure 3b). The mRNA levels of NFAT5 were also detected in ox-LDL treated cells models transfected miR-486-5p mimic or not, and high expression of NFAT5 was detected in cell models, which was decreased by miR-486-5p mimic transfection (Figure 3c).

## Discussion

CAS is a common atherosclerotic disease that commonly occurs in middle-aged and elderly people [19]. The current screening methods for CAS include digital subtraction angiography (DSA), transcranial Doppler ultrasound (TCD), magnetic resonance angiography (MRA), CT angiography (CTA), etc. In recent years, miRNA, as a novel biomarker, has been widely reported in various human diseases [20]. In CAS, aberrant expression of several miRNAs has been detected, such as miR-128-3p, miR-638, and miR-200c [10,21,22]. In the current study, asymptomatic CAS cases were enrolled, and the cases showed elevated levels of DBP, LDL, CRP, and ICAM-1. The findings reflected that CAS patients may be complicated with dyslipidemia and abnormal blood pressure, inflammatory reaction, and vascular endothelial injury, which may also be the inducements of CAS [23]. In addition, it was also found that the development of CAS is accompanied by the downregulation of serum miR-486-5p levels. And serum miR-486-5p was revealed to be an independent influence factor for the degree of CAS via logistic regression analysis.

MiR-486-5p has been revealed to have protective effects in cardiovascular and cerebrovascular diseases [17,24]. In patients with high blood pressure, significantly reduced miR-486-5p has been detected, which shows a close association with the cardiovascular risk score [17]. In addition, decreased exosomal miR-486-5p is related to antiangiogenesis in refractory intracranial atherosclerosis [24]. Moreover, microarray experiments also show the downregulation of miR-486-5p in coronary atherosclerotic plaque [16]. Consistently, the analysis results of clinical data in the current study proposed that serum miR-486-5p was negatively correlated with the levels of DBP, LDL, CRP, and ICAM-1 in CAS cases, suggesting its potential influence on the vascular endothelial injury, which supported its potential role in the progress of CAS. Furthermore, the ROC curve revealed its diagnostic ability to identify CAS cases from healthy controls.

Subcutaneous deposition and oxidative modification of LDL are the initial events of atherosclerosis [25]. OxLDL can induce endothelial cell apoptosis and promote the secretion of inflammatory factors and adhesion factors, leading to the accumulation of cholesterol in macrophages and the conversion of cholesterol into foam cells, ultimately causing the formation of atherosclerotic plaques. Endothelial cell damage is the initiating pathological basis of arteriosclerosis, and runs through the whole process of arteriosclerosis development. The dysfunction of vascular endothelial cells and activation of the inflammatory response ultimately cause the occurrence of CAS [3]. In the current study, HAECs were recruited for cell function experiments and treated with oxLDL. The results showed that oxLDL-induced HAEC apoptosis was weakened by increased miR-486-5p, reflecting its protective effect against vascular endothelial cell injury. Vascular endothelial cell injury promotes the release of intracellular adhesion factors and then induces the transformation of macrophages into foam cells [26]. The in vitro experimental results demonstrated that miR-486-5p can also inhibit the release of ICAM-1 in HAECs. Besides its regulatory role in the apoptosis of vascular endothelial cells, the present findings also demonstrated the influence of miR-486-5p on inflammation and oxidative stress. It is known that inflammation and oxidative stress are the main factors that induce vascular endothelial cell dysfunction. According to the present findings, oxLDL treatment contributed to inflammatory response and oxidative stress of HAECs; however, theses adverse impact caused by ox-LDL was reversed by miR-486-5p overexpression. In the previous studies, miR-486-5p has been widely reported to regulate inflammation and oxidative stress in several diseases, which was consistent with the findings in HAECs [27,28]. The present findings led us to hypothesize that miR-486-5p can prevent vascular endothelial dysfunction by inhibiting inflammation and oxidative stress, thus inhibiting the process of CAS.

NFAT5 is the main transcription factor activated by elevated osmotic pressure in mammalian cells, which can protect cells from hypertonic stimulation. NFAT5 plays an important role in inflammatory diseases and autoimmune function [29]. NFAT5 can promote arterial smooth muscle cell proliferation and motility in vitro, thus preventing atherosclerosis [30]. In addition, NFAT5 can also induce vascular endothelial cell apoptosis and inflammatory response, further contributing to the formation of atherosclerosis and CAS [31,32]. As previous evidence reported, NFAT5 is a target gene of miR-486-5p [33]. In the current study, the prediction of the binding between miR-486-5p and NFAT5 was done using bioinformatics analyses and verified through the dual-luciferase reporter assay. Furthermore, NFAT5 was overexpressed in ox-LDL treated HAECs, and the levels were downturned by increased miR-486-5p. The underlying mechanism was raised that miR-486-5p might be involved in the progress of CAS via targeting NFAT5.

## Conclusion

In conclusion, findings from the current study revealed that miR-486-5p was aberrantly underexpressed in CAS, and it might be able to be a promising biomarker for the early diagnosis of CAS. MiR-486-5p can prevent endothelial dysfunction, and the mechanism might be related to anti-inflammation and anti-oxidative via targeting NFAT5. This study provides potential therapeutic targets and ideas for the prevention and treatment of endothelial dysfunction and CAS.

## Supplementary Material

Supplemental Material
